# 

**Published:** 1800

**Authors:** 


### MEDICAL FACTS

AND

### OBSERVATIONS.

VOLUME THE EIGHTH.

Surgeon Genl's Office
LIBRARY.
Washington D.C.

LONDON:

PRINTED FOR J. CALLOW, CROWN COURT, SOHO;

BY W. SMITH, KING STREET, SEVEN DIALS.

M,DCCC.

1800

*Royal College of Physicians in Dublin, and M. R. I. A. From the Transactions of the Royal Irish Academy.* — — 215

XXIII. *History of a Case in which very uncommon Worms were discharged from the Stomach; with Observations thereon. By* Samuel Crumpe, *M. D. M. R. I. A. From the same Work.* — — 229

*Index.** — — — 239

DIRECTIONS TO THE BINDER.

Plate the First, (the figures of which are explained in pages 16, 17, 18, 22, and 23) may be placed at page 16; and plate the second, (references to which will be found at pages 129, 142, 180, 181, 182, and 234) at page 129.

ERRATA IN VOL. VII.

Page 29, line 3, *for* "thought" *read* "I thought."
155, line 5, *for* "1793" *read* "1795."

### INDEX.

### A.

ABDOMEN, Cafe of a Wound penetrating the Cavity of, — — — 137
Abernethy, John, his Account of fome Particulars in the Anatomy of the Whale, — — 199
————————, his Obfervations on the lymphatic Glands of the Horfe, — — 205
————————, his Reafons for fuppofing the lymphatic Glands, in moft Animals, to be cellular, 206, 207
Africa, Caufes affigned for the great Mortality among Europeans, on the Coaft of, — 107, and feq.
Allen, Gabriel, his Experience of the good Effects of Peach Leaves in Cafes of Nephritis, 123
Amygdalus Perfica, See *Peach.*
Anafarca, Inftances of, after Intermittents, cured by Mercury and Squills, — — 92
—————, See *Mercury.*
Ani Prolapfus, Cafe of, — 163
————————, different Modes of Treatment, in Cafes of, fuggefted, — 171, 172

### B.

Bance Ifland, its Unhealthinefs accounted for, 61

Barlow, John, his Mode of Practice in Cafes of diftorted Pelvis, in pregnant Women, — 185

Baudamont, M. Cafe of hairy Concretions found in the human Stomach, — — 145

Bell, Dr. James, Cafe of Retroverfion of the Uterus, 32

Bifhop, Sir William, his Account of the Effects of a Decoction of Peach Leaves, in Haematuria, 122

Brafs Veffel, See *Cassada.*

Calomel,

C.

Calomel, good Effects of, in fome Difeafes of Infants, 219, 221, 222
Caffada, Sweet, See *Jatropha.*
———————, Effects of fome, which had ftood in a brafs Veffel — — — 100
Cathrall, Dr. Ifaac, Cafe of ruptured Uterus, 146
Clarke, Dr. John, his Account of a Cafe of Monftrofity referred to, — — 13
Clarke, Dr. Jofeph, Remarks on fome Difeafes of Infancy, — — 215
Cold, Obfervations on the Influence of, upon the Health of the Inhabitants of London, — 208
Colic, epidemic, at Sierra Leone, Account of, 101, 102, 103
Convulfions, in early Infancy, Remarks on, 223
Coftivenefs, in Infants, how to be treated, 222
*Coup de Soleil*, mentioned as a rare Occurrence at Sierra Leone, — — — 93
Crumpe, Dr. Samuel, Cafe of Worms difcharged from the Stomach, — — — 229
Cutaneous Eruptions, in Infants, Remarks on the Treatment of, — — 225
Cynanche parotidea, Inftances of, in Children, at Sierra Leone, — — 94

D.

Denman, Dr. his Opinion on the Subject of Retroverfion of the Uterus, quoted — 50
Diarrhœa, in Infants, Remarks on, -- 216
Dyfphagia, brought on by Lightning, Cafe of, 111
Dyfpnœa, Inftance of, relieved by full Vomiting, 106

E.

Elytrocele, or vaginal Hernia, Inftance of, 39

Extra-Uterine Geftation, See *Geftation.*

F.

Free Town, Sierra Leone, its Situation defcribed, 63
Fryer, Henry, Two Cafes of Hernia congenita, 131
———————, Cafe of imperforate Hymen, 133

Fryer, Henry, Cafe of Fungus from a Wound in the Ear, — — 135
———————— a Wound penetrating the Cavity of the Abdomen, — — 137
Fungus, from a Wound in the Ear, Cafe of, 135

G.

Gangrene of the Stomach, brought on by Lightning, Cafe of, — — 111
Generation, Obfervations relative to, 12, 13, 14
Geftation, extra-uterine, many of the fuppofed Inftances of, thought to be Cafes of ruptured Uterus, 162
Glands, See *Lymphatic Glands.*
Guinea Ships, the great Mortality among the Crews of, accounted for, — — 107

H.

Haematuria, good Effects of a Decoction of Peach Leaves in a Cafe of, — — 122
Haighton, Dr. his Experiments on Generation, referred to, — — 14
Haller, Baron, his Work " de Monftris," referred to, 7
Hairy Concretions, found in the human Stomach, Cafes of — — 139, 145
Heberden, Dr. William, Jun. his Obfervations on the Influence of Cold on the Health of the Inhabitants of London, — — 208
Hernia congenita, Two Cafes of — 131
Home, Everard, on the Structure of Hydatids, 193
Hunter, Dr. William, his Idea of a Retroverfion of the Uterus, — — 49
Hydatids, Obfervations on the Structure of, 193
Hymen, imperforate, Cafe of, — 133

I.

Jatropha Janipha, grows in Abundance at Sierra Leone, — 67
Imperforate Hymen, Cafe of, — 133
Infancy, Remarks on fome Difeafes of, 215
Itch, in early Infancy, Remarks on the Treatment of, 227

Jugular Vein, internal, Account of a Cafe in which it was divided, — — 23

L.

Labour, premature, circumftances in which it is recommended to be brought on, — 186
Larvæ of Infects, difcharged from the Stomach, 229
——————— different Parts of the Body, Inftances of, — 236, 237
Lightning, Cafe of Gangrene of the Stomach, brought on by it, — — 111
Lithotomy, Cafe of, attended with remarkable Circumftances — — 126
Liver, in Infants, larger, in proportion to the Weight of the Body, than in Adults, — 220
Lymphatic Glands, fuppofed to be of a cellular Structure, 205
———————, Obfervations on the Structure of, in the Horfe, — — 206

M.

Meconium, Obfervations relative to it, — 7
————, the Opinion of its being fæces not tenable, 8
————, Colour of, how affected by Acids, 9, 10
Mercury, Inftances of anafarcous Patients who bore the Ufe of it well, without its fpeedily exciting Salivation, — — 92
Monro, Dr. his Account of a Cafe of Monftrofity, referred to, — — 8
Monftrofity, in a Child, Cafe of, — 1
Moon, Facts relative to the fuppofed Influence of, in Fevers, — — 89
Mumps, See *Cynanche Parotidea.*
Mufquitoes, not always Signs of an unhealthy Country, 65

O.

Ophthalmia, Obfervations relative to, 101

Paterfon,

P.

Paterſon, Patrick, Caſe of Gangrene of the Stomach, brought on by Lightning, — 111

Peach Leaves, good Effects of a Decoction of, in Haematuria, — — 122

Pelvis, diſtorted, Mode of Practice recommended in Caſes of, in pregnant Women, — 185

Premature Labour, See *Labour*.

Prolapſus Ani, Caſe of, — 163

R.

Rectum, a particular Operation on it ſuggeſted, in obſtinate Caſes of Prolapſus Ani, — 171

Retroverſion, of the Uterus, fatal Caſe of, 32

————————————, Obſervations on, 46

S.

Sankey, William, his Account of a Caſe of hairy Concretions found in the human Stomach, 140

Sierra Leone, Obſervations on the Climate and Diſeaſes of, — — — 56

Simmons, William, Caſe of Monſtroſity in a Child, 1

————————, his Deſcription of an improved ſcrew Tourniquet, — — 19

————————, his Account of a Diviſion of the internal jugular Vein, — 23

———————————————— Wound of the Uterus, — — — 26

Sims, Dr. John, Caſe of a ruptured Uterus, 146

————————, Mode of Treatment ſuggeſted by him in a Caſe of Prolapſus Ani, — 172

Singultus, Caſe of, — — 96

Stomach, Caſe of Gangrene of, brought on by Lightning, 111

—————, Caſes of hairy Concretions found in, 139, 145

Sun, Symptoms produced by long Expoſure to it, 93

T.

Tænia Hydatigena, a Species of Hydatid ſo called, 197

Teeth, ſupernumerary, Inſtance of a Swelling of the lower Jaw, and an Abſceſs in the Neck, occaſioned by, — — 173

Tobacco

Tobacco, Infufion of, its Effects in a Cafe of Anafarca, 92
Tourniquet, Hiftory of the Inftrument fo called, 19
———, as improved by Petit and Freke, defcribed, 20
———, an improved Screw, defcription of, 22
Turpentine, Enema of, its good Effects in Cafes of epidemic Colic, at Sierra Leone, — 103

U.

Vaginal Hernia, See *Elytrocele.*
Vein, internal jugular, Cafe in which it was divided, 23
Uterus, Retroverfion of, fatal Cafe of, 32
———, Obfervations on, 46
———, Cafe of a Wound of, that terminated favourably, 26
———, ruptured, Cafes of. — 146, 150

W.

Whale, fome Particulars in the Anatomy of, defcribed, 199
Whately, Thomas, his Account of the Appearances, on Diffection, of a ruptured Uterus, — 155
———, Cafe of prolapfus Ani, 163
———, Cafe of Abfcefs occafioned by fupernumerary Teeth, — — 173
Wickham, William, Cafe of Lithotomy, 126
Winterbottom, Dr. T. M. Obfervations on the Climate and Difeafes of Sierra Leone, — 56
Wood, William, Cafe of hairy Concretions found in the human Stomach, — — 139
Worms, difcharged from the Stomach, Cafe of, 229
———, fuppofed to be the Larvæ of fome Infect, — 234
———, faid to refemble the Larvæ of the common Beetle, — *ibid.*

END OF THE EIGHTH VOLUME.

Printed by W. Smith, King Street, Seven Dials, London.

